# Supplementary material for: Examining the human infectious reservoir for Plasmodium falciparum malaria in areas of differing transmission intensity
Source: Nat Commun. 2017 Oct 26;8:1133. doi: 10.1038/s41467-017-01270-4 (PMC5658399; doi:10.1038/s41467-017-01270-4)
Supplement: Supplementary file 1 — Supplementary Information [file 41467_2017_1270_MOESM1_ESM.pdf]

Supplementary Note 1

Sensitivity analysis on age-specific exposure to mosquitoes in Burkina Faso

To assess whether the non-negligible number of mosquitoes with multiple human source blood meal could have influenced our results, we fit mixed effects negative binomial models similar to the model described in the *Methods* section under different assumptions. In one model, we evenly allocated mosquito bites with multiple human DNA sources to all individuals living in the households where they were collected. Under this conservative assumption, adults (incidence rate ratio [IRR] 7.7 95% confidence interval [CI] 3.8 – 15.7) and children aged 5 to 15 years (IRR 3.7 95% CI 1.8 – 7.4) were more often bitten by *Anopheles* mosquitoes than younger children (**Supplementary Table 3**). In a different analysis, we assumed that individuals who had the lowest genetic distances to the genotypes present in these meals were blood sources. As most (98%) singly matched meals involved mean squared allelic distances lower than 1, this cut-off was used. Using this approach, we linked 125 multiple-source blood meals to one or two individuals in the same household, and we observed that adults (IRR 10.9 95% CI 4.6 – 25.8) and schoolchildren (IRR 4.6 95% CI 2.0 – 10.5) were more often exposed to anopheline vectors than children aged < 5 years.

Supplementary Note 2

Non-falciparum malarias and their potential infectiousness

Microscopically-detectable non-falciparum malaria parasites were present in all study sites. Patent *Plasmodium malariae* infections were more common in Mbita (5.9 [12/202] and 7.9 [16/202] % prevalence during dry and rainy seasons, respectively); only one individual in Kilifi had *P. malariae* co-infection. In Laye, three participants had patent malariae parasites, whilst in Balonghin one malariae infection was observed. *Plasmodium ovale* infections (N=4) were

also detected during surveys in Balonghin and Mbita (wet season). In total, 29 individuals had more than one malaria species identified in blood smear.

Overall, 8 of the 39 infectious individuals had patent non-falciparum malaria parasites (**Supplementary Table 1**). In Laye, one individual who infected 2/77 mosquitoes had patent malariae gametocytes and no evidence of falciparum infection by microscopy or molecular assays. In Balonghin, one individual who infected 17/35 mosquitoes had both patent ovale and falciparum gametocytes. In Mbita, 5/9 infectious individuals carried both patent falciparum and malariae parasites and one, only malariae parasites, including gametocytes.

Supplementary Note 3

Quantification of parasites

We observed a strong association between *18S* qPCR parasite densities and microscopy parasite densities. Where both assays detected parasites, microscopy overestimated parasite densities compared to *18S* qPCR (**Supplementary Figure 1**). One possible explanation for this overestimation is the assumption made during microscopy-based quantification that white blood cells levels are equal to 8,000 cell per µL. In one of the study sites in Burkina Faso (Balonghin), 34.4% of children recruited to a recent primaquine trial had white blood cell counts per µL of blood below 6,000 at enrolment (Gonçalves, personal communication).

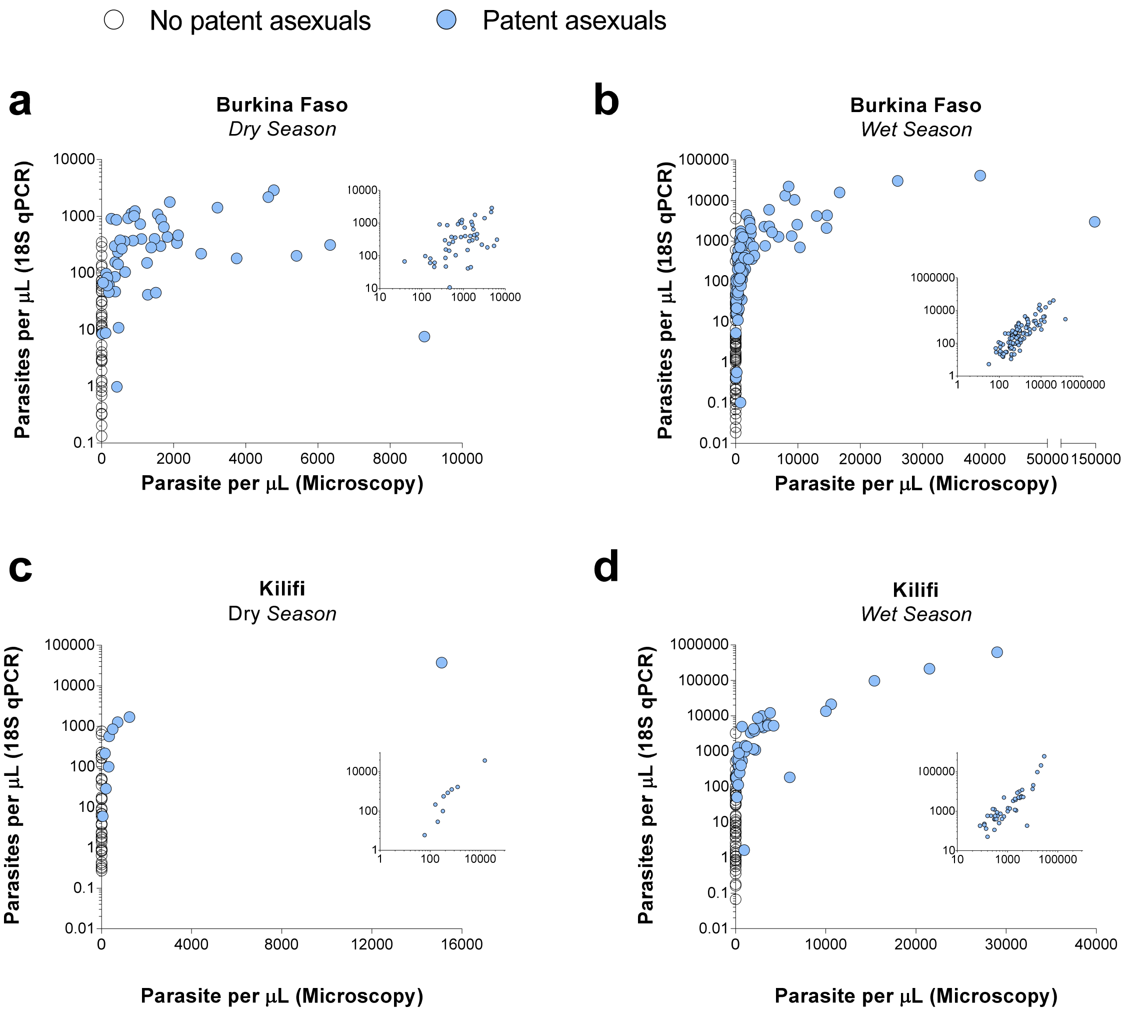

Supplementary Figure 1. Parasite quantification by *18S* rRNA gene qPCR and microscopy in samples collected in Laye (a) and Balonghin (b) and during Kilifi dry (c) and wet (d) season surveys. To facilitate visualisation, y-axes (parasite densities quantified by *18S* qPCR) are in $\log_{10}$ scale. Samples from four individuals with patent asexual stage parasites (one sample from Burkina Faso dry season survey, and three from Kilifi wet season survey) did not have parasites detected by *18S* qPCR and are not presented. The inset plots present parasitaemias when parasites were detected by both methods.

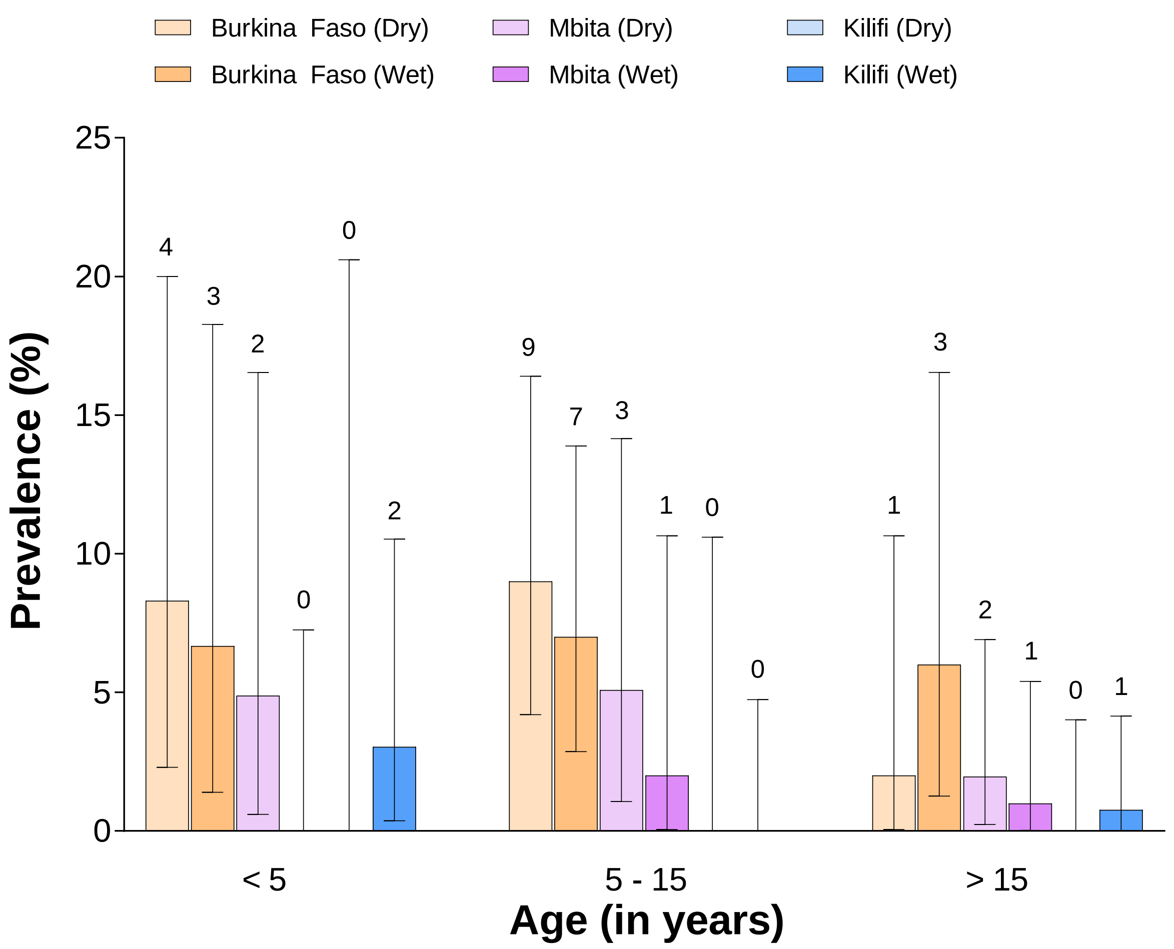

Supplementary Figure 2. Age-specific prevalences of infectiousness by site and season. The y-axis represents the percentage of individuals who were able to infect at least one mosquito in membrane feeding assays by age group and survey (x-axis); 95% exact binomial confidence intervals are shown. The number of infectious individuals in each age group and survey is presented above each bar.

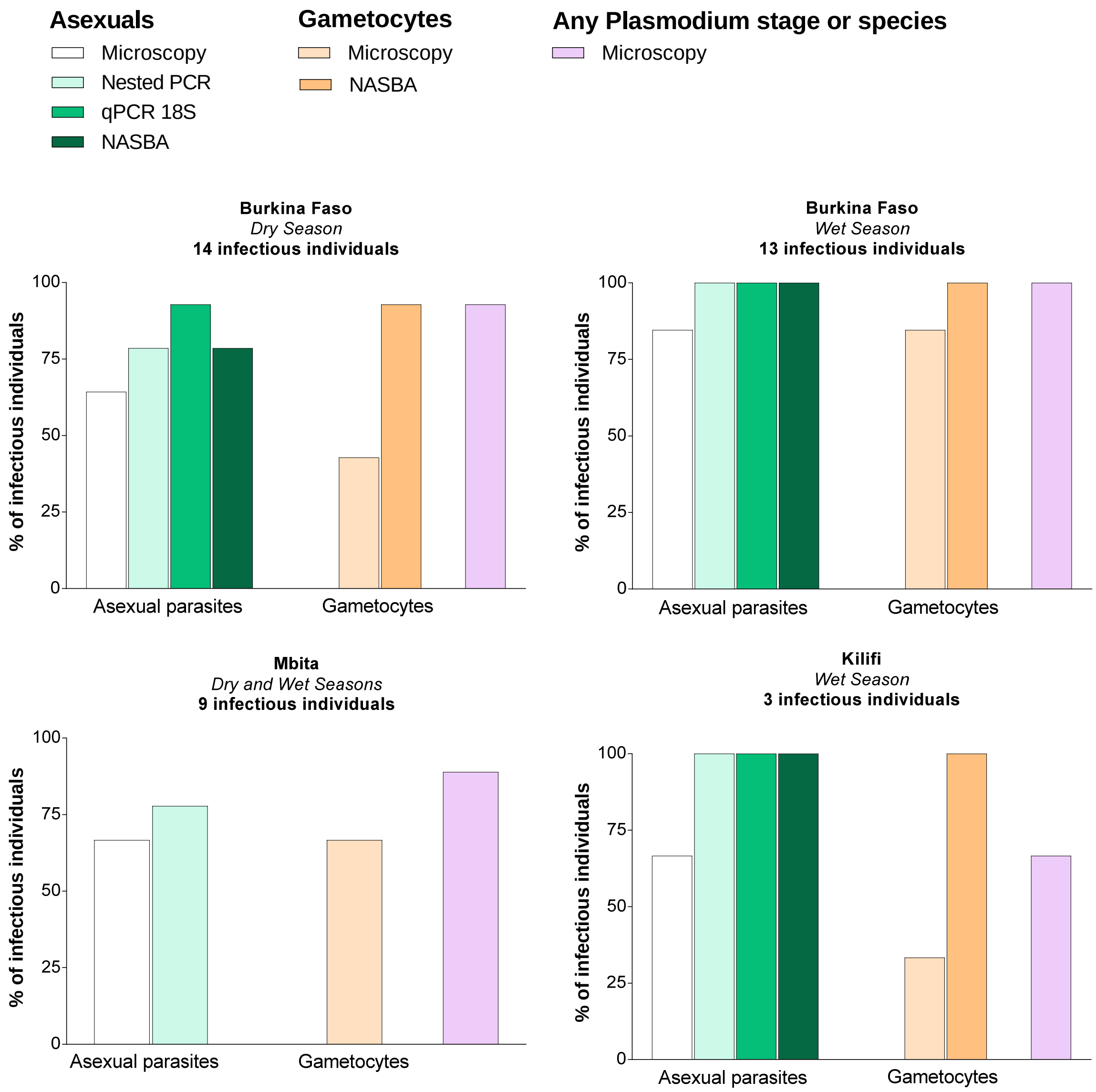

Supplementary Figure 3. Infectiousness to mosquitoes and infection detectability. The percentages (y-axes) of infectious individuals with parasites detected by different diagnostic methods (x-axes) are presented.

Supplementary Table 1. Infectious individuals sorted study site survey, and 18S qPCR-based parasite density.

Study Site	Survey	Proportion (n/N) infected mosquitoes	Median (range) oocyst count	Patent falciparum asexual	Patent falciparum gametocytes	*P. malariae* parasites	*P. ovale* parasites	*Pfs25* mRNA QT-NASBA	*18S* rRNA QT-NASBA	*18S* qPCR	*18S* qPCR *(parasites per µL)*	*Pfs25 QT-NASBA (gametocytes per µL)*	Molecular confirmation of falciparum mosquito infection
Laye	Dry	0.03 (2/77)	(1, 1)	Negative	Negative	Positive	Negative	Negative	Negative	Negative	0.0	0.0	Negative
Laye	Dry	0.10 (7/71)	3 (1 – 4)	Negative	Positive	Negative	Negative	Positive	Positive	Positive	0.4	110.1	All *P. falciparum*
Laye	Dry	0.27 (21/78)	2 (1 – 5)	Negative	Positive	Negative	Negative	Positive	Positive	Positive	0.7	9.2	All *P. falciparum*
Laye	Dry	0.04 (3/73)	1 (1 – 1)	Positive	Positive	Negative	Negative	Positive	Positive	Positive	1.0	7.2	All *P. falciparum*
Laye	Dry	0.05 (3/65)	1 (1 – 1)	Negative	Positive	Negative	Negative	Positive	Negative	Positive	8.6	10.8	All *P. falciparum*
Laye	Dry	0.16 (16/98)	1 (1 – 5)	Negative	Negative	Negative	Negative	Positive	Positive	Positive	11.9	10.9	All *P. falciparum*
Laye	Dry	0.02 (2/96)	(1, 3)	Positive	Negative	Negative	Negative	Positive	Positive	Positive	150.3	50.3	All *P. falciparum*
Laye	Dry	0.20 (14/71)	3.5 (1 – 11)	Positive	Negative	Negative	Negative	Positive	Positive	Positive	200.9	88.1	All *P. falciparum*
Laye	Dry	0.28 (24/87)	2.5 (1 – 22)	Positive	Positive	Negative	Negative	Positive	Positive	Positive	218.8	42.5	All *P. falciparum*
Laye	Dry	0.03 (3/88)	2 (1 – 2)	Positive	Positive	Negative	Negative	Positive	Positive	Positive	281.2	11.0	All *P. falciparum*
Laye	Dry	0.01 (1/97)	1	Positive	Negative	Negative	Negative	Positive	Positive	Positive	399.0	24.1	All *P. falciparum*
Laye	Dry	0.02 (2/81)	(1, 1)	Positive	Negative	Negative	Negative	Positive	Positive	Positive	431.5	13.0	All *P. falciparum*
Laye	Dry	0.01 (1/76)	1	Positive	Negative	Negative	Negative	Positive	Positive	Positive	1119.4	21.4	All *P. falciparum*
Laye	Dry	0.13 (11/84)	2 (1 – 4)	Positive	Negative	Negative	Negative	Positive	Negative	Positive	1782.4	54.3	All *P. falciparum*
Balonghin	Wet	0.03 (1/39)	1	Negative	Positive	Negative	Negative	Positive	Positive	Positive	0.4	21.2	All *P. falciparum*
Balonghin	Wet	0.05 (2/39)	(1, 2)	Negative	Positive	Negative	Negative	Positive	Positive	Positive	1.3	116.9	All *P. falciparum*
Balonghin	Wet	0.33 (14/43)	2 (1 – 5)	Positive	Positive	Negative	Negative	Positive	Positive	Positive	15.1	106.2	All *P. falciparum*
Balonghin	Wet	0.05 (2/44)	(2, 2)	Positive	Negative	Negative	Negative	Positive	Positive	Positive	54.1	68.1	All *P. falciparum*
Balonghin	Wet	0.14 (5/35)	2 (1 – 5)	Positive	Positive	Negative	Negative	Positive	Positive	Positive	114.3	89.9	All *P. falciparum*
Balonghin	Wet	0.03 (1/33)	1	Positive	Positive	Negative	Negative	Positive	Positive	Positive	356.5	32.7	All *P. falciparum*
Balonghin	Wet	0.34 (17/50)	3 (1 – 16)	Positive	Negative	Negative	Negative	Positive	Positive	Positive	435.5	161.1	All *P. falciparum*
Balonghin	Wet	0.29 (15/51)	1 (1 – 15)	Positive	Positive	Negative	Negative	Positive	Positive	Positive	450.8	144.5	All *P. falciparum*
Balonghin	Wet	0.49 (17/35)	4 (1 – 15)	Positive	Positive	Negative	Positive	Positive	Positive	Positive	712.9	0.04	Negative
Balonghin	Wet	0.23 (8/35)	4 (2 – 13)	Positive	Positive	Negative	Negative	Positive	Positive	Positive	1324.3	162.5	All *P. falciparum*
Balonghin	Wet	0.27 (12/45)	4 (1 – 9)	Positive	Positive	Negative	Negative	Positive	Positive	Positive	2264.6	220.8	All *P. falciparum*
Balonghin	Wet	0.50 (24/48)	16 (1 – 71)	Positive	Positive	Negative	Negative	Positive	Positive	Positive	4305.3	3228.5	All *P. falciparum*
Balonghin	Wet	0.07 (3/41)	8 (3 – 8)	Positive	Positive	Negative	Negative	Positive	Positive	Positive	30478.9	144.9	All *P. falciparum*
Mbita	Dry	0.07 (4/60)	1 (1 – 1)	Negative	Negative	Negative	Negative	-	-	-	-	-	1 mosq. *P. falciparum*
Mbita	Dry	0.02 (1/56)	1	Positive	Negative	Positive	Negative	-	-	-	-	-	-
Mbita	Dry	0.57 (17/30)	8 (2 – 197)	Negative	Positive	Positive	Negative	-	-	-	-	-	1 mosq. *P. malariae*
Mbita	Dry	0.02 (1/44)	3	Positive	Positive	Positive	Negative	-	-	-	-	-	-
Mbita	Dry	0.02 (1/60)	1	Positive	Positive	Negative	Negative	-	-	-	-	-	-
Mbita	Dry	0.02 (1/60)	1	Positive	Positive	Negative	Negative	-	-	-	-	-	-
Mbita	Dry	0.05 (3/60)	1 (1 – 1)	Positive	Positive	Positive	Negative	-	-	-	-	-	1 mosq. *P. malariae*
Mbita	Wet	0.07 (2/30)	(1, 2)	Negative	Negative	Positive	Negative	-	-	-	-	-	
Mbita	Wet	0.08 (3/37)	2 (1 – 4)	Positive	Positive	Positive	Negative	-	-	-	-	-	-
Kilifi	Wet	0.07 (1/15)	1	Negative	Negative	Negative	Negative	Positive	Positive	Positive	30.7	2.0	Positive
Kilifi	Wet	0.04 (2/45)	(1, 4)	Positive	Positive	Negative	Negative	Positive	Positive	Positive	1361.4	266.1	Positive
Kilifi	Wet	0.03 (1/32)	3	Positive	Negative	Negative	Negative	Positive	Positive	Positive	5345.8	107.9	Positive

Supplementary Table 2. Malaria diagnostics and contribution to the local population of infectious individuals.

	Contribution to the pool of infectious individuals (%)		
	Laye	*Balonghin*	*Kilifi (Wet Season)*
**Diagnostics**			
No patent parasites and PCR +	5.7	0	46.5
< 100 parasites per µL	35.2	41.5	0.0
100 - 200 parasites per µL	0.0	4.3	0.0
> 200 parasites per µL	59.0	54.1	53.5

Supplementary Table 3. Sensitivity analyses of age-specific mosquito exposure in Burkina Faso.

	Incidence Rate Ratio (95% confidence interval)		
	*Excluding mixed blood meal mosquitoes**	*Including mixed blood meal mosquitoes and assuming equal exposure***	*Including mixed blood meal mosquitoes and matching to lowest genetic distance*
**Age (in years)**			
< 5	Reference	Reference	Reference
5 – 15	7.7 (2.9 - 20.8)	3.7 (1.8 - 7.4)	4.6 (2.0 - 10.5)
> 15	20.9 (7.7 - 57.4)	7.7 (3.8 - 15.7)	10.9 (4.6 - 25.8)

*as reported in the main text; based on 666 uniquely matched mosquitoes

**assuming equal exposure among age groups of mosquitoes with multiple blood meals. This analysis was based on 666 singly matched mosquitoes and 153 mosquitoes with multiple human DNA sources that were allocated evenly to all individuals living in the households where they were collected.

Supplementary Table 4. Distribution of multiple source blood meals by number of loci with multiple alleles (three or more alleles per loci)

	**Study sites**	
**Number of loci with multiple alleles (3 or more)**	*Balonghin*	*Mbita*
3	45	27
4	29	13
5	38	21
6	28	8
7	11	12
8	2	2
9	0	4
10	0	0
